# FLASH Proton Pencil Beam Scanning Irradiation Minimizes Radiation-Induced Leg Contracture and Skin Toxicity in Mice

**DOI:** 10.3390/cancers13051012

**Published:** 2021-03-01

**Authors:** Shannon Cunningham, Shelby McCauley, Kanimozhi Vairamani, Joseph Speth, Swati Girdhani, Eric Abel, Ricky A. Sharma, John P. Perentesis, Susanne I. Wells, Anthony Mascia, Mathieu Sertorio

**Affiliations:** 1 Cincinnati Children’s Hospital Medical Center, Division of Oncology, Cincinnati, OH 45229, USA; Shannon.Cunningham@cchmc.org (S.C.); Shelby.McCauley@cchmc.org (S.M.); Kanimozhi.Vairamani@ppd.com (K.V.); John.Perentesis@cchmc.org (J.P.P.); Susanne.Wells@cchmc.org (S.I.W.); 2 Department of Radiation Oncology, University of Cincinnati College of Medicine, Cincinnati, OH 45229, USA; Joe.Speth@UCHealth.com (J.S.); Anthony.Mascia@cchmc.org (A.M.); 3 Varian Medical Systems, Inc., Palo Alto, CA 94304, USA; Swati.Girdhani@varian.com (S.G.); Eric.Abel@varian.com (E.A.); ricky.sharma@ucl.ac.uk (R.A.S.); 4 Department of Pediatrics, University of Cincinnati College of Medicine, Cincinnati, OH 45229, USA

**Keywords:** FLASH, ultra-high dose rate, proton therapy, proton beam scanning, skin and soft tissue, normal tissue toxicity

## Abstract

**Simple Summary:**

Dose and efficacy of radiation therapy are limited by the toxicity to normal tissue adjacent to the treated tumor region. Recently, ultra-high dose rate radiotherapy (FLASH radiotherapy) has shown beneficial reduction of normal tissue damage while preserving similar tumor efficacy with electron, photon and scattered proton beam irradiation in preclinical models. Proton therapy is increasingly delivered by pencil beam scanning (PBS) technology, and we therefore set out to test PBS FLASH radiotherapy on normal tissue toxicity and tumor control in vivo in mouse using a clinical proton delivery system. This validation of the FLASH normal tissue-sparing hypothesis with a clinical delivery system provides supporting data for PBS FLASH radiotherapy and its potential role in improving radiotherapy outcomes.

**Abstract:**

Ultra-high dose rate radiation has been reported to produce a more favorable toxicity and tumor control profile compared to conventional dose rates that are used for patient treatment. So far, the so-called FLASH effect has been validated for electron, photon and scattered proton beam, but not yet for proton pencil beam scanning (PBS). Because PBS is the state-of-the-art delivery modality for proton therapy and constitutes a wide and growing installation base, we determined the benefit of FLASH PBS on skin and soft tissue toxicity. Using a pencil beam scanning nozzle and the plateau region of a 250 MeV proton beam, a uniform physical dose of 35 Gy (toxicity study) or 15 Gy (tumor control study) was delivered to the right hind leg of mice at various dose rates: Sham, Conventional (Conv, 1 Gy/s), Flash60 (57 Gy/s) and Flash115 (115 Gy/s). Acute radiation effects were quantified by measurements of plasma and skin levels of TGF-β1 and skin toxicity scoring. Delayed irradiation response was defined by hind leg contracture as a surrogate of irradiation-induced skin and soft tissue toxicity and by plasma levels of 13 different cytokines (CXCL1, CXCL10, Eotaxin, IL1-beta, IL-6, MCP-1, Mip1alpha, TNF-alpha, TNF-beta, VEGF, G-CSF, GM-CSF and TGF- β1). Plasma and skin levels of TGF-β1, skin toxicity and leg contracture were all significantly decreased in FLASH compared to Conv groups of mice. FLASH and Conv PBS had similar efficacy with regards to growth control of MOC1 and MOC2 head and neck cancer cells transplanted into syngeneic, immunocompetent mice. These results demonstrate consistent delivery of FLASH PBS radiation from 1 to 115 Gy/s in a clinical gantry. Radiation response following delivery of 35 Gy indicates potential benefits of FLASH versus conventional PBS that are related to skin and soft tissue toxicity.

## 1. Introduction

Conventional X-ray radiation therapy (XRT) remains one of the major therapies for cancer, and half of all cancer patients receive XRT treatments. Total deliverable dose to the tumor has always been a limiting factor due to radiation-induced toxicity to adjacent normal tissues and organs. Proton therapy (PT) features physics characteristics that allow for increased tumor control and decreased toxicities. While X-ray irradiation deposits energy continuously along the beam path, charged particles deposit most of their energy at the end of their trajectory in the so-called Bragg peak, without exit dose and with lower dose deposition along the path of the beam. Thus, PT generally allows maximal dose deposition in the tumor while reducing off-target dose deposition in normal tissue. Even with the high accuracy of more recent proton beam scanning (PBS) radiotherapy, however, tissues proximal or adjacent to the tumor target will still receive off-target dose deposition. In most clinical scenarios, skin and muscle are inevitable organs at risk for any type of radiotherapy. Despite this improvement of normal tissue toxicity by PT, complications are therefore still observed during the course of patient treatment, for the most part causing skin and soft tissue damage [1,2,3,4,5].

Since 2014, growing interest in further improvements have emerged in the radiation oncology field based on the observation that ultra-high dose rate electron radiation delivery (average dose rate > 40 Gy/s), referred to as FLASH, showed less toxicity to normal tissue despite similar tumor control in comparison to conventional proton dose rates (0.5–1 Gy/s) used in the clinic [6]. Radiation-induced skin fibrosis and muscle atrophy are often observed in patients with head and neck squamous cell carcinoma (HNSCC) and breast cancer [7,8]. Skin complications range from acute dermatitis to long-term reactions such as ulceration and/or skin fibrosis. Muscle damage is also frequently observed after radiotherapy treatment and includes muscular pain and long-term definitive muscle atrophy. Skin fibrosis along with muscle atrophy ultimately leads to a stiffening of the affected area, creating issues with facial appearance, motion range limitations, discomfort and chronic pain. A mouse model based on the measurement of radiation-induced leg contracture has been extensively used to study and develop preventive treatments for radiation-induced skin/soft tissue toxicity [9,10,11]. Radiation-induced fibrosis (RIF) is a complex cascade of events starting with tissue damage and acute inflammatory responses which leads to the activation of myofibroblasts responsible for the remodeling of the extracellular matrix [12]. Pathological fibrosis after radiation is the consequence of a deregulated tissue healing process due to sustained oxidative stress and cytokines, including transforming growth factor beta 1 (TGF-β1) [13].

FLASH dose delivery using electron, X-ray and more recently scattered protons to animal models was reported to improve toxicity of a range of normal tissues including skin, while retaining similar tumor control [6,14,15,16,17,18]. Recent trends in proton therapy demonstrate a growing interest and usage of pencil beam scanning (PBS) technology [19,20]. Studies of the FLASH effect for PBS have yet to be reported. Moreover, the combination of the spatial dose delivery advantage to the tumor and minimal FLASH effects on normal tissue might be a promising window of opportunity towards improving radiotherapy treatment outcomes.

Validation of the FLASH effect for PBS radiotherapy is important given the different dose delivery properties which may impact the FLASH effect compared to previously used radiation modalities. Contrasting with a uniform but pulsed radiation field from photon or electron modalities or a continuous and uniform dose and fluency field from scattered protons, PBS fields contain hundreds or thousands of discrete spots that together create uniform fields. Simply put, only a portion of the targeted area receives radiation at any given time. Because the main theory behind the FLASH effect relies on decreased reactive oxygen species (ROS) generation after radiation, perhaps due to local oxygen depletion [21], and because PBS dose rates are already higher (0.5–1 Gy/s) than X-ray or electron dose rates, it is not known whether PBS will provide a similar benefit for the total tumor area targeted at an average FLASH dose rate. At the current time, only transmission treatment using the plateau area of a mono energy proton beam can be achieved. Spread-Out Bragg Peak (SOBP) treatment at FLASH dose rates still requires further technological development [22]. SOPB treatment is based on succession of layer of beam spots allowing precise dose delivery to the tumor volume. SOBP requires modulation of the energy of the proton beam during the treatment delivery in order to create different layers of beam spots. For the purposes of this study, we studied single-layer, transmission fields at FLASH dose rates. This is a viable method for future FLASH radiotherapy clinical trials, even if the ultimate modality may be Bragg Peak FLASH radiotherapy. In the Bragg Peak FLASH radiotherapy case, validating the FLASH effect on the entrance region is still valuable as, for most deep-seated tumors, skin, soft tissue and other organs may be in the beam path and therefore irradiated.

For this purpose, we used a classical radiation-induced mouse leg contracture assay to define toxicity of FLASH versus conventional PBS on skin and soft tissue for the first time. We used a Varian ProBeam clinical gantry at the CCHMC/UC proton center to conduct the work described in this manuscript. We were therefore able to deliver up to 35 Gy at an average dose rate of 1 Gy/s and up to 115 Gy/s. Improvement of leg contracture and skin toxicity was observed in mice receiving PBS irradiation at FLASH dose rates compared to conventional dose rates (1 Gy/s). Because other studies have shown dysregulation of inflammation and cytokine levels after FLASH irradiation in different tissues [21,23,24], we defined FLASH PBS tumor control in vivo using a syngeneic mouse model. MOC1 and MOC2 cells, originally derived from oral carcinoma that arose in C57Bl/6 mice [25], formed subcutaneous tumors on the right hind leg of immunocompetent mice. In accordance with other radiation modalities, FLASH PBS was equivalent to conventional PBS for the control of indolent (MOC1) and aggressive (MOC2) tumors in the context of a functional immune system. Taken together, we demonstrate the existence of a beneficial FLASH effect on skin and soft tissue using proton PBS, and show that FLASH PBS is equivalent to conventional dose rate for the control of indolent and aggressive squamous cell carcinoma.

## 2. Results

### 2.1. FLASH Dose Rate Is Achievable Using the Clinical PBS Gantry System

**Figure 1:**
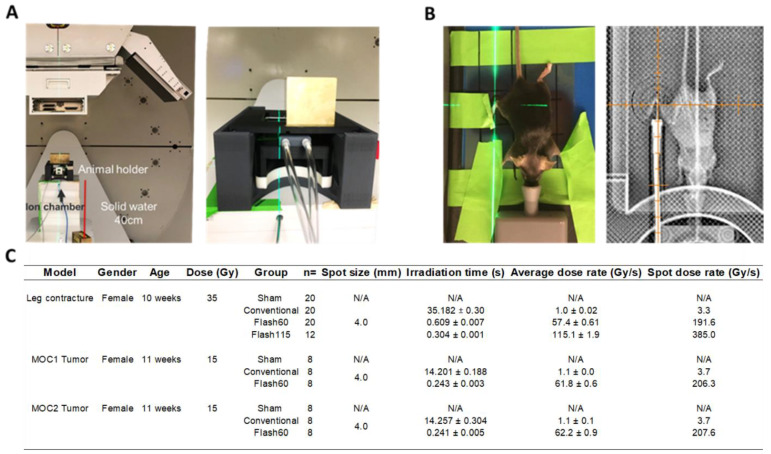
Irradiation approach for PBS radiation of the murine hind leg. (**A**) Representative picture of the animal holder, ion chamber and beam blocker set up in the proton gantry used for all irradiations. (**B**) Representative pictures of the alignment strategy, showing the mouse being first positioned on gafchromic film in the removable holder bed and validation of alignment by the gantry X-ray system. (**C**) Description of beam delivery parameters for the different mouse groups and experimentations. Values are expressed as the average ± SD. PBS: pencil beam scanning.

To precisely align and deliver proton radiation to the right hind leg of mice with or without engrafted tumors, we used an in-house 3D-printed mouse immobilizer and jig allowing anesthesia of the animals on a removable bed outside the main mouse holder. The mouse holder was maintained in the same position during the entire experiment (Figure 1A,B). Field quality control, dose, dose rate and animal positioning were confirmed before irradiation as described in materials and methods. Using this set-up, we delivered 35 Gy and 15 Gy in a single fraction at an average dose rate of 1.0 Gy/s (Conv), 57.4 Gy/s (Flash60) and a maximum achievable dose rate of 115.1 Gy/s (Flash115) with a variation of dose rate ranging from 0.9% to 1.9% (Figure 1C). Using the Varian ProBeam proton clinical gantry system, we were able to reproducibly and stably deliver PBS radiation at average dose rates above 40 Gy/s. The described set-up was used to determine the benefit of FLASH dose rate in a mouse model of skin and soft tissue toxicity.

### 2.2. TGF-β1 Production Is Attenuated Following FLASH PBS versus Conventional Radiation

**Figure 2:**
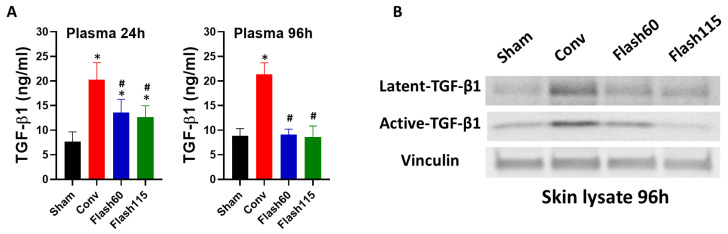
Decreased early TGF-Β1 production following FLASH PBS versus Conv irradiation. Right hind legs of the mice were PBS-irradiated with 35 Gy or left untreated (Sham) with dose rates of 1 Gy/s (Conv), 57 Gy/s (Flash60) and 115 Gy/s (Flash115). (**A**) Plasma measurement of total TGF-Β1 by ELISA at 24 and 96 h post irradiation of the mice at the different indicated dose rates (*n* = 8/group). (**B**) Detection of latent (45 kD) and activated (12 kD) TGF-β1 by Western blot analysis in individual sham or irradiated mouse leg skin at 96 h post treatment is shown. Vinculin was used as a loading control. Differences between conditions were determined by a one-way ANOVA test with a multiple-comparison Tukey correction. * *p* ≤ 0.05 vs. Sham and # *p* ≤ 0.05 vs. Conv. Bars represent the mean +/− SD. The whole western blot figures can been see in the Supplementary Materials.

To measure the effect of FLASH versus conventional PBS on skin and soft tissue toxicity, the right hind leg of C57BL/6 female mice was irradiated. Mice were randomly separated into groups and irradiated with a total of 35 Gy at either 1 Gy/sec (Conv), 57 Gy/s (Flash60) or 115 Gy/sec (Flash115). TGF-β1 has been widely reported as a marker of tissue damage, and causes radiation-induced fibrosis in lung and skin tissue [26,27]. Moreover, TGF-β1 levels are known to increase in skin and blood in the acute phase of the radiation-induced stress response [28,29,30]. Therefore, we measured systemic and local levels of TGF-β1 in sham or PBS-irradiated mice at day 1 (blood) and day 4 (blood and skin) post radiation in order to quantify the acute radiation response (Figure 2). As expected, Conv irradiation induced an increase of TGF-β1 level in the blood at day 1, and this increase was sustained at day 4 post IR (Figure 2A). On day 1, TGF-β1 levels in the Flash60 and Flash115 groups were above Sham but below Conv levels. On day 4, TGF-β1 levels in those same groups were reduced down to baseline. Decreased latent and active TGF-β1 levels were also observed in the irradiated skin on day 4 for the FLASH groups compared to Conv (Figure 2B). Thus, FLASH PBS versus Conv leads to attenuated TGF-β1 production following radiation, and this might indicate a diminished radiation-induced stress response in both blood and skin.

### 2.3. Diminished Leg Contracture and Skin Toxicities in Response to FLASH PBS versus Conventional Radiation

**Figure 3:**
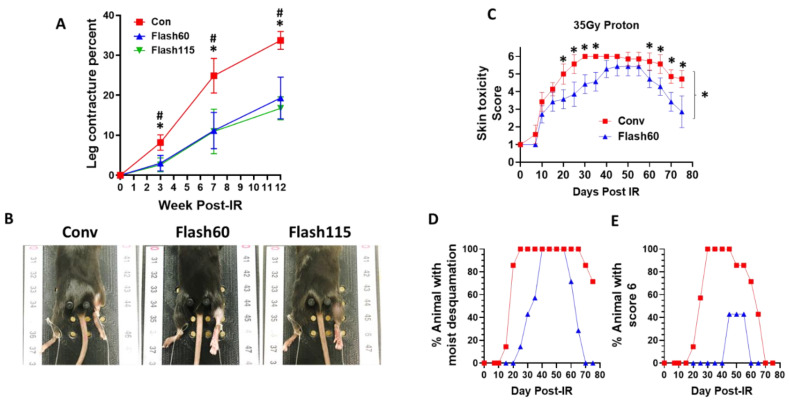
Benefits of FLASH PBS with regards to leg contracture and skin toxicity. Right hind legs of the mice were either PBS-irradiated with 35 Gy or left untreated (Sham) with dose rates of 1 Gy/s (Conv), 57 Gy/s (Flash60) and 115 Gy/s (Flash115). (**A**) Irradiated leg contracture measurements at 3, 7 and 12 weeks post irradiation. Leg contracture was calculated as described in Materials and Methods using the non-irradiated left leg of each animal as a reference (*n* = 8/group). (**B**) Representative pictures of leg extension measurements with an in-house 3D-printed jig at 84 days post IR. (**C**) Irradiated skin toxicity score of Conv (red square) and Flash60 (blue triangle) mouse groups (*n* = 8/group) as a function of time after radiation. Skin toxicity was scored as: Score 1 = normal, Score 2 = alopecia, Score 3 = erythema, Score 4 = dry desquamation, Score 5 = <30% moist desquamation and Score 6 = >30% moist desquamation. (**D**,**E**) Percentage of mice with (**D**) moist desquamation (scores 5 + 6) or (**E**) irradiated leg moist desquamation >30% of irradiated area (score 6) after Conv (red square) and Flash60 (blue triangle) irradiation. Differences between conditions were determined by a two-way ANOVA test with multiple-comparison Tukey correction. Bars represent mean +/− SD. * *p* ≤ 0.05 vs. Flash60 and # *p* ≤ 0.05 vs. Flash115.

As a surrogate for radiation-induced skin and soft tissue toxicity, we quantified murine leg contracture over a period of 12 weeks post irradiation. As expected, animals in all groups developed contracture of the irradiated compared to the non-irradiated leg over time (Figure 3A,B). However, FLASH dose rate groups harbored significantly decreased contractures in comparison to Conv mice as early as 3 weeks post irradiation and up to the end of the experiment. No significant differences were observed between the Flash60 and Flash115 group. A second, independent cohort of mice was shaved 72 h before irradiation and used for skin toxicity studies comparing Flash60 with Conv radiation. As no difference had been observed between Flash60 and Flash115 on leg contracture, we focused on comparison of Conv vs. Flash60 effects on skin toxicity to prevent unnecessary use of animals. Skin toxicity was quantified based on a previously published score [29] ranging from normal (score 1) to severe moist desquamation (>30% irradiated area, score 6). Compared to Conv, Flash60 PBS irradiation induced a lesser degree of toxicity with a reduced skin damage score (Figure 3C). Furthermore, animals in the Flash60 group exhibited a significant delay in moist desquamation, together with accelerated resolution (Figure 3D,E). Only 47% of the Flash60-treated animals developed the highest level of moist desquamation (score 6) between days 45 and 55 in comparison with 100% of Conv-treated animals between days 30 and 55. Thus, FLASH PBS versus Conv irradiation offers the advantage of less severe soft tissue damage and skin toxicity over Conv treatment.

### 2.4. Detection of Limited Number of Cytokine Level Changes in the Blood of FLASH-Irradiated Mice

**Figure 4:**
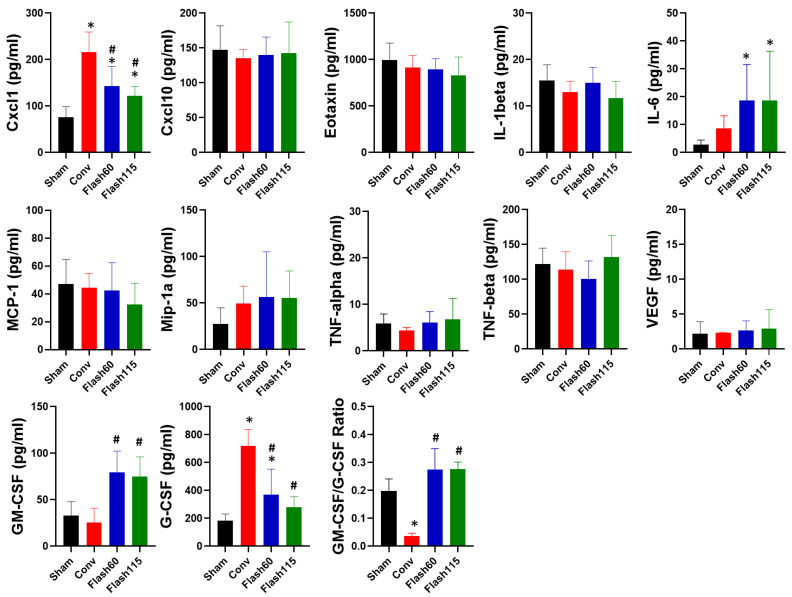
Cytokine response to FLASH PBS- versus Conv-treated mice. Right hind legs of the mice were PBS-irradiated with 35 Gy or left untreated (Sham control mice) with dose rates of 1 Gy/s (Conv), 57 Gy/s (Flash60) and 115 Gy/s (Flash115). Blood was collected at 12 weeks post radiation and the levels of 12 cytokines were quantified by multiplex array (*n* = 8/group). Differences between conditions were determined by a one-way ANOVA test with multiple-comparison Tukey correction. * *p* ≤ 0.05 vs. Sham and # *p* ≤ 0.05 vs. Conv. Bars represent mean +/− SD.

To detect systemic markers of FLASH PBS in the blood of the irradiated mice, we measured 12 different cytokines (CXCL1, CXCL10, Eotaxin, IL1-beta, IL-6, MCP-1, Mip1alpha, TNF-alpha, TNF-beta, VEGF, G-CSF and GM-CSF) by multiplex array at 12 weeks post radiation (Figure 4). Among these cytokines, only Cxcl-1, G-CSF and GM-CSF were significantly different between the Conv- and FLASH-irradiated groups. Cxcl-1 and G-CSF levels were increased in Conv- versus FLASH-irradiated animals, while GM-CSF was decreased. Published reports using blood from patients with cystic fibrosis have demonstrated that the GM-CSF over G-CSF ratio is inversely correlated with the degree of tissue toxicity [31]. In line with increased toxicity in response to Conv (Figure 3), Conv-treated animals had a decreased GM-CSF/G-CSF score when compared to FLASH PBS- or Sham-treated animals. IL-6 levels were significantly increased in FLASH animals in comparison to SHAM control animals but not in Conv animals. No significant differences were observed between Flash60- and Flash115-treated animals for any cytokines. Thus, FLASH PBS versus Conv treatment regulates a distinct subset of cytokines in the blood of irradiated animals, which may reflect and perhaps promote a lesser degree of toxicity.

### 2.5. Conv and FLASH PBS Exhibit Equivalent Control of Tumor Growth in Immunocompetent Mice

**Figure 5:**
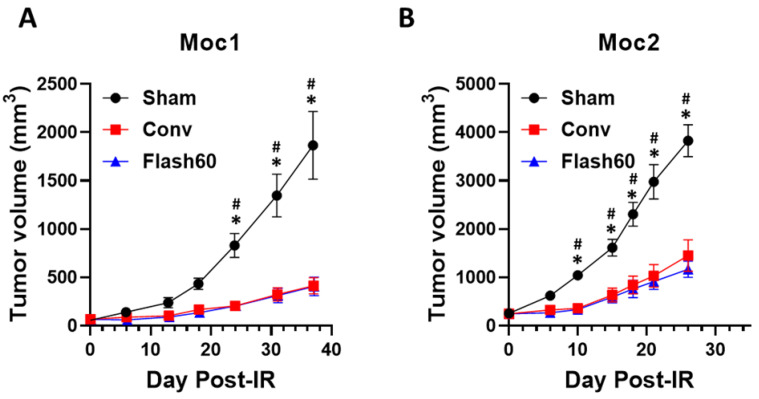
FLASH PBS and Conv irradiation induce equivalent control of indolent and aggressive squamous cell carcinomas. MOC1 (2 × 10^6^ cells) or aggressive MOC2 (2 × 10^5^ cells) murine squamous cell carcinoma cells were injected subcutaneously into the right flanks of C57Bl/6 immunocompetent mice (*n* = 8/group). Twenty-one days post injection, the mice were randomized in groups of equivalent average tumor sizes. Tumors were irradiated with 15 Gy of Conv or FLASH PBS. (**A**) MOC1 and (**B**) MOC2 tumor volume measurements as a function of post irradiation time. Differences between conditions were determined by a two-way ANOVA test with multiple-comparison Tukey correction. Bars represent mean +/− SD. * *p* ≤ 0.05 vs. Conv and # *p* ≤ 0.05 vs. Flash60.

To determine whether FLASH tumor control was retained in the face of reduced toxicity, we quantified treatment-induced regression of indolent (MOC1 cell-derived) and aggressive (MOC2 cell-derived) murine squamous cell carcinomas in a well-established immunocompetent mouse model (Figure 5). This allows definition of radiation efficacy in an immunocompetent mouse background. To generate tumors, MOC1 or MOC2 cells were injected subcutaneously into the right hind leg of C57bl/6 mice to quantify radiation efficacy in the same area that was used for toxicity studies. After 3 weeks, the mice were separated into 3 groups of equal average tumor size (MOC1: Sham = 69.4 ± 20.9 mm^3^, Conv = 66.7 ± 19.3 mm^3^, Flash60 = 68.6 ± 17.5 mm^3^ and MOC2: Sham = 128.5 ± 39.1 mm^3^, Conv = 125.0 ± 35.1 mm^3^, Flash60 = 124.9 ± 36.8 mm^3^) and either treated with 15 Gy Conv or FLASH PBS or left untreated (Sham). Both Conv- and FLASH-irradiated groups exhibited a significant tumor growth delay in comparison to the Sham animals. After both dose rate modalities, tumors grew less robustly at 18 days post radiation for MOC1 cells (Figure 5A) and at 10 days post radiation for MOC2 cells (Figure 5B). No differences were observed between the Conv and Flash60 dose rates at different time points. Taken together, FLASH PBS versus Conv treatment offers equivalent control of tumor growth with significantly fewer toxicities.

## 3. Discussion

The FLASH effect has been reported previously in vivo in murine preclinical models with electron [6], photon [32] and scattered proton beams [14]. However, a similar effect for proton pencil beam scanning (PBS) has not yet been described, yet is key for future development of FLASH PBS. The differences in dose delivery comparing the available clinical radiotherapy modalities suggest the need to validate the FLASH effect in PBS itself. Given that the FLASH mechanistic hypotheses imply that the FLASH effect is dependent on specific time structures, it is not a given that different modalities or delivery systems at the same dose and dose rate yield the same FLASH effect. Further investigations into the spatial and temporal structures of PBS and their impact on the FLASH effect are warranted because of the growing interest in PBS application in clinic [19,20]. We conducted the first investigation of FLASH PBS consequences on skin and soft tissue using a clinical gantry with no modification of the beam line. The gantry nozzle was equipped with an updated Varian FLEX ion chamber to ensure stable delivery of conventional and FLASH dose rate with no impact on radiation. With respect to dose rate, several definitions exist in the literature along with their clinical implications [33]. For this study, composite or average dose rate is used as the comparator. Relative comparisons demonstrating differential effects using differing dose rates with the same radiation delivery modality are at liberty to use one of several dose rate definitions with no impact on the differential effect results. However, caution must be exercised when comparing results from other modalities (e.g., electron or non-scanning protons). For example, the cumulative and local dose rate for pencil beam scanning may be a factor of 5–10 or more different; whereas, no such distinction exists for passive scattering protons. Settling the important issue of how to compare different modalities with respect to dose rate or a dose rate-related parameter definition is beyond the scope of this study. Composite dose rate is defined as the total field dose divided by the total field irradiation time [33]. This is the most conservative definition of the published dose rates, thus ensuring that a minimum FLASH dose rate condition is guaranteed. For this study, modification of the beam current (i.e., higher current for FLASH, lower current for conventional) is the only change when switching between dose rate cohorts. This ensures accurate comparison of different conditions among the same delivery modality by removing potential issues from differential set-up or radiation source between conventional or FLASH dose rates. This clinical gantry set-up is likely to accelerate future translation of the pre-clinical data to the clinic by removing extra validation steps for experimental delivery systems. Our PBS gantry delivery system was validated for a larger field size (8 cm × 8 cm) and delivery of 8 Gy at an average dose rate of 60 Gy/s (data not shown). Those FLASH radiation conditions are currently used at the CCHMC/UC Proton Center in a human feasibility study (https://www.clinicaltrials.gov/ct2/show/NCT04592887 (accessed on 27 February 2021)).

Our initial focus was on the consequences of FLASH versus conventional PBS dose rates on skin and soft tissues. This choice was motivated, in part, by the fact that skin and soft tissues are inevitably organs at risk of most radiotherapy procedures and they are the normal tissues exposed to any incident irradiation. To this end, we used a classical, well-defined irradiation-induced leg contracture mouse model as a surrogate for skin and soft tissue damage [9]. This model is based on the measure of the shortening of the irradiated rear limb of the mice caused by radiation damage to the skin and soft tissue. In response to FLASH versus control PBS irradiation, we observed reduced skin toxicity and leg contracture. The improved mouse skin toxicity at FLASH dose rate is in accordance with a recent study using electron in the same dose range with a dose rate of 180 Gy/s [17]. Because leg contracture was identical between 57 and 115 Gy/s average dose rates, it appears that maximum FLASH effect was already achieved at the average dose rate of 57 Gy/s. The optimal FLASH dose rate in this study is different than in a previous report showing maximal FLASH effect on mouse brain at a dose rate of 100 Gy/s. This could indicate a difference between normal tissue origins on dose rate requirement for optimal FLASH effect. A second explanation might relate to the fact that the dose rate at the beam spot is higher than the average field dose rate, and therefore a maximal FLASH effect might be obtained at a lower average dose rate in comparison to other scattered radiation modalities. The latter possibility would have significant implications for the design of future clinical trials along with technological development of the delivery system.

We confirmed previously reported in vitro data wherein a reduction of TGF-β1 production was reported after FLASH irradiation [34]. Early TGF-β1 level reduction observed in the blood and skin may be indicative of a lesser degree of initial tissue damage following FLASH PBS. Reduced initial stress is also supported by the attenuated development of acute skin damage along with a reduced proportion of animals developing maximal skin toxicity. This is in line with published studies showing that inhibition of TGF-β1 signaling improves acute skin toxicity in mice [35]. Reduction of early tissue damage after FLASH dose rate irradiation was also observed by others as a reduction of initial DNA damage or induction of oxidative stress [21,36]. Less TGF-β1 production in response to FLASH PBS might have a long-term benefit on the development of the contracture phenotype since TGF-β1 is in part responsible for stimulation of the inflammatory response post irradiation and activation of fibroblast to a pro-fibrotic phenotype [13]. Such attenuated inflammation has been demonstrated with electron and X-ray FLASH irradiation in the lung and electron FLASH irradiation in the brain [24,32]. Further investigation of the induction and duration of inflammation in this model could deepen our insights into benefits (and potentially risks) of FLASH PBS on radiation-induced skin and soft tissue toxicities.

To determine potential sustained effects of FLASH versus conventional PBS irradiation, we measured the levels of a panel of 12 cytokines at 12 weeks post irradiation in the blood. Among these cytokines, CXCL1 levels were increased in the Conv-irradiated animals in comparison to both FLASH animal groups. Interestingly, increased levels of CXCL1 have been reported in the blood of UVB-irradiated mice, and inhibition of CXCL1 was sufficient to improve UVB-induced skin toxicity. Furthermore, reducing UVB-induced skin toxicity in mice using quercetin and astragalin was correlated with reduced level of CXCL1 in protected animals. Thus, reduced CXCL1 in the FLASH PBS versus Conv-treated mice might support the attenuated skin toxicity observed in FLASH PBS- versus Conv-treated animals. Interestingly, differences in G-CSF and GM-CSF levels and the GM-CSF/G-CSF ratio were also observed in the FLASH animal groups in comparison to the Conv group. Observations in the blood of patients with cystic fibrosis showed a correlation between elevated G-CSF/GM-CSF ratios and a lesser degree of lung damage. In line with those observations, functional characterization of G-CSF and GM-CSF have shown that while both cytokines were able to activate neutrophils, only G-CSF was able to attract neutrophils [37] which are also elevated in irradiation-induced skin dermatitis [12]. G-CSF but not GM-CSF levels were correlated with high neutrophil numbers in the broncho-alveolar lavage of acute respiratory distress syndrome patients [38]. Thus, increased G-CSF levels and a decreased GM-CSG/G-CSF ratio observed in Conv-treated mice supports the increased level of skin damage observed in this group at 12 weeks. Increased levels of IL-6 that are only observed in FLASH animal groups are in line with reduced skin toxicity and accelerated recovery. Previous studies have shown that IL-6 was an important immunomodulatory signal essential for the repair of UV radiation damage to the skin through induction of IL-10 [39] and for limiting dermatitis following irritant contact through deregulation of IL-22R expression [40]. Importantly, we did not observe a global change of cytokines in this set between the FLASH and Conv groups of mice, indicating the dysregulation of specific cytokines, which remain to be further investigated in response to FLASH with regards to their activities and timed modulation. Overall, the differential pattern of a subset of cytokines observed for FLASH PBS is in accordance with previous work and implicates the inflammatory response in the FLASH effect. The benefit of FLASH RT on inflammatory response has been observed in mouse brain [23] and mini-pig skin [18] with different radiation sources and thus could be a common important mechanism of the FLASH sparing effect. Only limited data are available concerning the tumor inflammatory response following FLASH RT. To our knowledge, only one group has shown preliminary data suggesting increased tumor lymphocyte recruitment following proton FLASH RT [41]. Nevertheless, those results also point to an immunomodulatory effect of FLASH RT at the tumor levels. Thus, further studies need to be conducted to fully understand the effect of FLASH RT on normal tissue and tumor immune response. Because the Conv- and FLASH-irradiated animals exhibited differential levels of skin toxicity at 12 weeks, we cannot currently rule out the possibility that blood cytokines are regulated by, rather than the cause of, skin and/or soft tissue damage.

Because of the above FLASH versus Conv radiation-induced differential cytokine production with possible inflammatory responses, we quantified the relative efficacy of these radiation qualities on tumor regression in immunocompetent mice. We used the immuno-active indolent MOC1 and immuno-cold aggressive MOC2 squamous cell carcinoma flank tumor model. Those 2 cell lines were derived from oral squamous cell carcinoma (OSCC) tumors from immunocompetent C57Bl/6 mice and are commonly used to study squamous cell carcinoma tumor growth and treatment in an immunocompetent background [25,42,43]. MOC1 is considered an indolent model and MOC2 an aggressive model with metastatic capacity. Interestingly, those 2 cell lines have been extensively characterized genetically, and harbor driver mutations similar to those in human OSSC [44], reinforcing their authenticity for pre-clinical studies. To be consistent with single-radiation dose use in previous FLASH radiation studies (8–34 Gy) [45] and based on previous irradiation studies of in vivo MOC2 tumor [43], we used a single dose of 15 Gy to study the FLASH versus Conv tumor control efficacy. This choice was also motivated by our experience with MOC1 tumors reaching a complete growth arrest or shrinkage above proton irradiation doses of 15 Gy (data not shown). FLASH and Conv proton PBS had similar efficacy in both tumor models. This shows the specificity of FLASH-specific PBS tissue-sparing effect on normal tissue as observed with other radiation modalities. The similarity in tumor control between two different cell lines suggests that, as a single agent, FLASH PBS does not confer additional tumor kill in comparison to Conv proton PBS. This is only based on quantification of tumor growth, since modification of the microenvironment was not measured here. Further investigation of the tumor microenvironment and molecular response to FLASH remains to be carried out. The MOC1/2 models could help define the impact of FLASH on the tumor microenvironment based on stromal and immune composition of the tumors. Because the leg contracture assay and levels of different cytokines measured in this study did not show differences between Flash60 and Flash115 irradiation and to avoid the unnecessary use of animals, we decided to limit the tumor control efficacy comparison to Conv versus Flash60. We cannot rule out the possibility that Flash115 might be more or less effective for tumor control. However, previous studies [6,36,46,47] have shown similar tumor control efficacy across different Flash dose rates.

A limitation of this study is the usage of single-fraction radiation doses. The 35 Gy single-fraction dose is commonly used for quantification of skin toxicity and leg contracture and effects of therapeutic drugs on both phenotypes. This single high-dose regimen is far from conventional fractionation treatment used in clinic. There is a current need to verify the existence of a FLASH effect with dose and fractionation regimen relevant for clinical applications and we take the first required step towards addressing this question using the experimental set-up developed in this study. Use of the mouse model could be a limiting factor to further investigate precise proton PBS irradiation delivery to a small volume of normal tissue or precise targeting of the tumor to recapitulate clinical practice. Because this study was done with a clinical gantry that is utilized for human treatment, larger animal models such as rats or veterinary trials could easily be conducted with the same delivery system. Finally, the use of just one mouse strain is a limitation. Radiation sensitivity due to mouse strain-dependent DNA damage repair, immune response and development of tissue injury is well documented and this work needs to be extended to a broader range of mouse strains. This might deepen our understanding of the FLASH tissue-sparing effect, especially with regards to differences in inflammatory responses as observed in this study and reported in previous studies using different radiation modalities [18,23].

## 4. Materials and Methods

### 4.1. Proton Delivery, Dosimetry and Monitoring

The ProBeam Pencil Beam Scanning Gantry (Varian Medical Systems, Palo Alto, CA, USA) was used to deliver a monoenergetic, single-layer transmission radiation field. FLASH dose rates were delivered at 250 MeV, while conventional dose rates were delivered at 244 MeV. Since the water-equivalent depth of the mouse is small (i.e., approximately 1.0 cm), the small change in incident energy does not yield a measureable difference in linear energy transfer or material stopping power at the mouse depth. Full CW (72 MHz) beam currents of 1.7 nA, 90 nA and 180 nA were used to obtain dose rates of 1 Gy/s, 57 Gy/s and 115 Gy/s respectively.

The field dosimetric metrology system is composed of both an ion chamber and electrometer and radiochromic film and scanner. Ion chamber and electrometer calibration factors have been determined independently by an Accredited Dosimetry Calibration Laboratory (ADCL). The radiochromic film and scanner are cross-calibrated to the ion chamber. Using the International Atomic Energy Agency (IAEA) TRS-398 absolute dose formalism, collection efficiency and recombination effects are quantified, using two-voltage technique, and the ion chamber correction is measured to be less than 1.0% in all conditions. Independently, the ion chamber was validated against a graphite calorimeter in order to ensure accuracy of a dose and dose-rate independence. The ion chamber and graphite calorimeter agree within 1.0%. Fields were measured for flatness and symmetry using calibrated gafchromic film (Gafchromic EBT3) and calibrated flatbed scanner (Epson 10000XL). Furthermore, fields were measured for absolute dose and dose rate using a calibrated Advanced Markus (PTW, Freiburg im Breisgau, Germany) ion chamber connected to a calibrated IBA Dose1 (IBA-Dosimetry, Schwarzenbruck, Germany) electrometer. Tolerances of 5% flatness and symmetry, 3% for dose and 5% for dose rate, were used. The single-layer spot patterns were designed using in-house software to a 5% uniform dose of 35 Gy or 15 Gy to a field of 25 mm × 23 mm (95% isodose line) composed of 30 individual spots (beam spot sizetion = 4.0 mm) with a 0 degree gantry position. The field was irradiated with a continuous beam without beam pause between the spots. Prior to irradiation, the dose was measured at a water equivalent depth of 1.0 cm in a solid water phantom using a calibrated Advanced Markus ion chamber (PTW Germany) connected to a calibrated IBA Dose 1 electrometer (IBA Dosimetry Germany). Dose rate was determined by computing the ratio of the total dose to total field delivery time as provided by the machine log files. During irradiation, an Advanced Markus ion chamber was placed distal to the mouse to verify the dose for each irradiation. To ensure proper alignment, the mouse jig was first positioned at isocenter using the gantry laser alignment system (accuracy 1 mm). The radiation field alignment was visually verified by irradiating gafchromic film secured within the jig. Each mouse was placed such that the leg was coinciding with the isocenter using both the laser system and visual indication from the gafchromic film. Localization was verified on a subset of mice using the orthogonal kilovoltage image guidance system, and no discrepancies with the laser alignment were detected.

### 4.2. Irradiation-Induced Leg Contracture and Skin Toxicity

All animal studies were approved by the Cincinnati Children’s Hospital Institutional Animal Care and Use Committee. For IR-induced leg contracture, the unshaved right hind leg of 10-week-old female C57Bl/6j mice was aligned to the proton beam in a 3D in-house-printed jig equipped with an anesthesia nose cone to ensure animal anesthesia during the treatment with a Somnosuite unit using 2.5% isoflurane and ambient air. The room video system was used to monitor animal immobility. After alignment validation by the gantry X-ray imaging system, the right hind leg was irradiated with an absolute dose of 35 Gy at conventional dose rate of 1 Gy/s (Conv, *n* = 12 mice/group) or at FLASH dose rate of 57 Gy/s (Flash60, *n* = 12 mice/group) or 115 Gy/s (Flash115, *n* = 12 mice/group). At 96 h post irradiation, 4 mice/group were euthanized in order to collect the skin of the irradiated (right) or Sham (left) rear hind for protein extraction. The remaining 8 mice per group were checked twice a week to ensure the absence of severe morbidity. Leg contracture of the mice was measured using a 3D-printed jig at 3, 7 and 12 weeks post irradiation. The un-irradiated (Sham) left hind leg of each mouse was used as control to calculate the leg contracture score. The extension of the sham (left) and irradiated (right) leg was measured under a 0.1 N force. Percentage leg contracture was calculated as: [1 − (leg extension irradiated leg/leg extension sham leg)] × 100.

For radiation-induced skin toxicity, the right hind leg of an independent cohort of mice was shaved 72 h before radiation to allow accurate scoring of early skin toxicity. Eight animals per group were irradiated as described above. Skin toxicity was graded every 5 days until the end of the study at 12 weeks. A previously published skin toxicity grade scale was used [29]: Score 1 = normal, Score 2 = alopecia, Score 3 = erythema, Score 4 = dry desquamation, Score 5 =<30% moist desquamation and Score 6 =>30% moist desquamation.

### 4.3. Irradiation Efficacy on Tumor Growth Control

Eight-week-old female C57Bl/6j mice per group were injected subcutaneously into their shaved right hind leg with either 2 × 105 MOC2 or 2 × 106 MOC1 mouse oral carcinoma cell lines in PBS [25]. Three weeks post injection, tumors were measured with calipers and mice were randomized using an Excel macro function to create 3 groups of equal average tumor size. The tumor-bearing hind legs were irradiated as described above with an absolute dose of 15 Gy and dose rate of 1 Gy/s (Conv) or 60 Gy/s (Flash60). Tumor volume was determined by caliper measurement once a week post irradiation. Volume was calculated as Tumor volume (mm^3^) = (Width tumor^2^ (mm) × Length tumor (mm))/2

### 4.4. Cytokine Blood Level Quantification

A volume of 150 uL of blood from isoflurane anesthetized mice (*n* = 8 mice/group) was collected into EDTA micro tubes (Becton Dickinson, Bergen County, NJ, USA) via retro orbital bleeding on day 1, 4, and 84 post irradiation with 35 Gy Conv or FLASH PBS. Plasma was collected by 15 min centrifugation at 2000× *g* and kept frozen at −80 °C until cytokine measurement. Total TGF-β1 levels were measured using the LEGEND MAX mouse TGF-β1 ELISA kit (BioLegend, San Diego, CA, USA) following the manufacturer’s protocol. Plasma from the 12 week time point was also used to measure murine CXCL1, CXCL10, Eotaxin, G-CSF, G-MCSF IL-1beta, IL-6, MCP-1, MIP-1alpha TNF-alpha, TNF-beta and VEGF by Luminex cytokine array. The Luminex measurements were done by the CCHMC Research Flow Cytometry Core following the manufacturer’s recommendation. Plasma samples (5 uL) were mixed with 25 uL cytokine array beads overnight in reaction plates. After 2 washes, 25 uL of detection antibody were added to each sample and incubated for 1 h at room temperature. After 1 h incubation, 25 uL of S-RPE were added directly to the samples for 30 min. Plates were washed and 150 uL of sheath fluid were added. Samples were analyzed using a Luminex 200 dual-laser system.

### 4.5. Western Blot Analysis

Skin from the right hind leg of Sham or irradiated animals was harvested 4 days post irradiation and kept frozen at −80 °C before processing. Total protein extraction was done by bead beating using a FastPrep-24 classic bead beating grinder (MP Biomedical, Santa Ana, CA, USA) in RIPA buffer containing HALT protease and phosphatase inhibitor (Thermo Scientific, Waltham, MA, USA). Protein concentrations were quantified by BCA assay (Thermo Scientific). TGF-β1 was then detected by Western blot analysis. Briefly, 20 ug of protein were separated on 4–20% SDS-Page gradient gel (BioRad, Hercules, CA, USA) and transferred onto PVFD membrane for immuno-detection using a primary rabbit polyclonal anti-TGF-β1 antibody (Genetex, GTX130023) or a primary rabbit polyclonal anti-Vinculin antibody (Genetex, GTX109749) and a secondary HRP-linked anti-rabbit antibody (Cytiva, Marlborough, MA, USA). Chemi-luminescence signal was acquired using a Chemidoc device (Bio-Rad).

### 4.6. Statistical Analysis

Statistical analysis was performed using GraphPad Prism v8.0.1. One-way ANOVA test with Tukey correction for multiple comparison was used for analysis of data in Figure 2A and Figure 4. Two-way ANOVA test with Tukey correction for multiple comparison was used for analysis of data in Figure 3A,B and Figure 4A,B. Differences between groups of mice were considered significant for *p* ˂ 0.05. All data are represented as mean ± standard deviation (SD).

## 5. Conclusions

In conclusion, we report the benefit of FLASH PBS on normal skin and soft tissue toxicity using a clinical proton PBS gantry delivery system with no experimental modification. The irradiation set-up developed in this study can be easily adapted for other animal models and for body areas or organs of interest for radiotherapy such as brain or lung. Also, the use of a clinical PBS delivery system is key for translation of FLASH PBS using current delivery systems into future clinical trials.

## Data Availability

The data presented in this study are available on request from the corresponding author. The data are not publicly available due to a disclosure agreement with Varian Medical System Inc.

## Supplementary Materials

The following are available online at https://www.mdpi.com/2072-6694/13/5/1012/s1, Supplementary Figures: The whole western blot figures for Figure 2b.
