# Supplementary figures and images for: FLASH Proton Pencil Beam Scanning Irradiation Minimizes Radiation-Induced Leg Contracture and Skin Toxicity in Mice
Source: Cancers (Basel). 2021 Mar 1;13(5):1012. doi: 10.3390/cancers13051012 (PMC7957631; doi:10.3390/cancers13051012)

Original western blot images used for Figure2.B

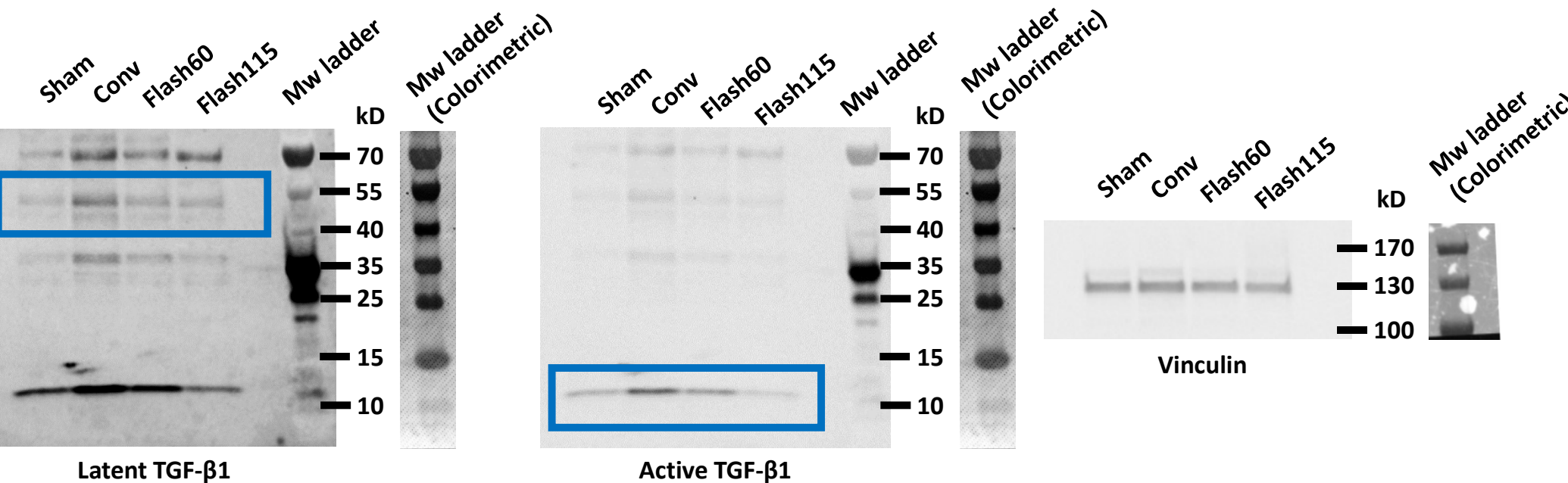

Supplement: Supplementary file 1 [file cancers-13-01012-s001.pdf]
